# Yeast as a Model to Find New Drugs and Drug Targets for *VPS13*-Dependent Neurodegenerative Diseases

**DOI:** 10.3390/ijms23095106

**Published:** 2022-05-04

**Authors:** Joanna Kaminska, Piotr Soczewka, Weronika Rzepnikowska, Teresa Zoladek

**Affiliations:** 1 Institute of Biochemistry and Biophysics Polish Academy of Sciences, 02-106 Warsaw, Poland; kaminska@ibb.waw.pl (J.K.); psoc@ibb.waw.pl (P.S.); 2 Neuromuscular Unit, Mossakowski Medical Research Institute, Polish Academy of Sciences, 02-106 Warsaw, Poland; wrzepnikowska@imdik.pan.pl

**Keywords:** yeast, chorea-acanthocytosis, VPS13, VPS13A-D, calcium signalling, copper homeostasis, iron homeostasis

## Abstract

Mutations in human *VPS13A-D* genes result in rare neurological diseases, including chorea-acanthocytosis. The pathogenesis of these diseases is poorly understood, and no effective treatment is available. As *VPS13* genes are evolutionarily conserved, the effects of the pathogenic mutations could be studied in model organisms, including yeast, where one *VPS13* gene is present. In this review, we summarize advancements obtained using yeast. In recent studies, *vps13*Δ and *vps13-I2749* yeast mutants, which are models of chorea-acanthocytosis, were used to screen for multicopy and chemical suppressors. Two of the suppressors, a fragment of the *MYO3* and *RCN2* genes, act by downregulating calcineurin activity. In addition, *vps13*Δ suppression was achieved by using calcineurin inhibitors. The other group of multicopy suppressors were genes: *FET4,* encoding iron transporter, and *CTR1*, *CTR3* and *CCC2*, encoding copper transporters. Mechanisms of their suppression rely on causing an increase in the intracellular iron content. Moreover, among the identified chemical suppressors were copper ionophores, which require a functional iron uptake system for activity, and flavonoids, which bind iron. These findings point at areas for further investigation in a higher eukaryotic model of *VPS13*-related diseases and to new therapeutic targets: calcium signalling and copper and iron homeostasis. Furthermore, the identified drugs are interesting candidates for drug repurposing for these diseases.

## 1. Introduction

According to European Union law, a disease is considered rare when it affects no more than 1 person per 2000. However, with around 7000 identified rare diseases, together they affect 3.5–10% of the population, which corresponds to approximately 30 million people in Europe and 300 million around the world [1]. A multitude and variety of rare diseases pose huge diagnostic difficulties. It often takes several years from occurrence of symptoms to the correct diagnosis of a rare disease [2,3,4,5,6,7,8,9]. Despite recent advances, such as new-generation sequencing (NGS), the time and accuracy of diagnosis of rare diseases have not much improved. Identifying a pathogenic mutation among thousands of detected single-nucleotide polymorphisms in NGS results is challenging. Functional studies of pathogenic mutations could be performed in laboratory model organisms, and yeast proved to be particularly important [10,11,12,13,14].

Another obstacle that the patients face is a great limitation of therapies. For a significant majority of rare diseases, current treatment is only symptomatic and focuses on improving the quality of patients’ life; however, the disease itself is not cured. Therefore, there is a great need to find new drug targets and drugs for rare diseases. Drug targets can be easily identified in yeast models by genetic methods, such as second-site suppressor screens and multicopy suppressor screens, which indicate where to hit to overcome observed defects. The costs of launching a new drug to the market may reach up to several billions of dollars, and the rare disease market is very limited [1]. In order to ease the market introduction of a drug for a rare disease, one can apply a drug repurposing approach. Drug repurposing aims to find new indications of drugs that are already approved for use in humans for other diseases [1]. Repurposed drugs can be directly tested in human clinical trials, even with low numbers of patients [1,15]. Drug repurposing is therefore an attractive and reasonable approach to rare diseases that promises the fast development of new therapies.

Here, we summarize attempts to improve our understanding of the pathogenesis and to identify potential therapeutic targets and repurposable drugs for rare diseases associated with mutations in *VPS13* (vacuolar protein sorting 13) genes using yeast as a model organism. Emphasis was given to recently published work on VPS13 proteins in the Special Issues of *IJMS* entitled “Yeast Models and Molecular Mechanisms of Neurodegenerative Diseases” and “Yeast Models and Molecular Mechanisms of Neurodegenerative Diseases 2021”.

## 2. *Saccharomyces cerevisiae* as a Disease Model and Simple Platform for High Throughput Screens

*S. cerevisiae* yeast, despite a simple, unicellular structure, is widely used for studying human diseases. Yeast models of neurodegenerative diseases [12,16,17], mitochondrial dysfunctions [18,19,20], metabolic disorders [21], ageing [16,22,23], prion diseases [24,25] or even cancer [26] were established. Modelling diseases in yeast is possible due to the evolutionary conservation of cellular physiology among eukaryotes [23,27] as well as the presence of homologous genes in human and yeast genomes—more than 6600 human genes have a corresponding yeast homologue [28], and some of them complement mutations in matching yeast genes. This allows yeast to be used for studying the effects of disease-causing mutations. One can either replace the yeast gene with a human allele carrying a pathogenic mutation—an example could be yeast models of copper metabolism diseases linked with mutations in the *ATP7A* and *ATP7B* genes resulting in Menkes and Wilson’s diseases, respectively [29,30,31]—or a mutation corresponding to a pathogenic human mutation could be introduced into a yeast gene in order to mimic an analogous change in protein functioning. For example, mutations in either mitochondrial or nuclear genes encoding mitochondrial enzymes are often modelled in yeast [14,18,20]. For a comprehensive view of the advantages of using yeast as a tool to assess the pathogenicity of mutations, read the review by Cervelli and Galli [32].

Modelling human diseases in yeast is possible even in cases when there are no orthologs in both species. Heterologous expression of a human gene can still influence the functioning of yeast cells. Examples are yeast models of neurodegenerative diseases with protein aggregation. Expression of genes encoding aggregating protein results in toxicity. In this manner, yeast models of Alzheimer’s disease (AD), Parkinson’s disease (PD) and Huntington disease (HD) have been developed, in which respective aggregating proteins, amyloid β, α-synuclein and huntingtin, are produced [16,33]. In cases when there are no obvious growth phenotypes, one can study the effects of heterologous expression of functional and pathogenic human alleles on yeast cellular physiology. The example here could be a yeast model of Charcot–Marie–Tooth disorder associated with the *GDAP1* gene [12]. While yeasts do not enable the investigation of a disease’s impact at the systemic level, they allow for studying the molecular pathology of a disease in a simple, fast and cost-effective way.

Once a yeast disease model is established, one can apply various research approaches that aim to discover new aspects of a disease pathogenesis and contribute to its treatment. One of the experimental approaches used in yeast models is screening for suppressors. A suppressor is a genetic or chemical factor that overcomes defects caused by a mutation. Among genetic suppressors, two classes can be distinguished: second-site and multicopy suppressors [34]. Multicopy suppressors are genes that, when overexpressed, overcome defects caused by mutations. This type of suppression can be achieved by: (I) improving complex stability due to the higher accessibility of one of the complex components; (II) increasing the pool of a defective complex with reduced activity; (III) increasing the activity of a defective pathway that has reduced activity; (IV) mitigating the toxicity of an upregulated pathway by increasing the pool of inhibitors or titrating activators of this pathway [34]. Importantly, high-throughput genetic suppressor screens are virtually impossible to be carried out in higher eukaryotic organisms due to the fact of their complex nature. Identifying a suppressor and understanding its mechanism of action could reveal new functional connections between a mutated gene and a suppressor. This may contribute to a better understanding of the molecular pathology of disease, uncover disease modulating genes and suggest novel therapeutic approaches [15,34].

**Figure 1:**
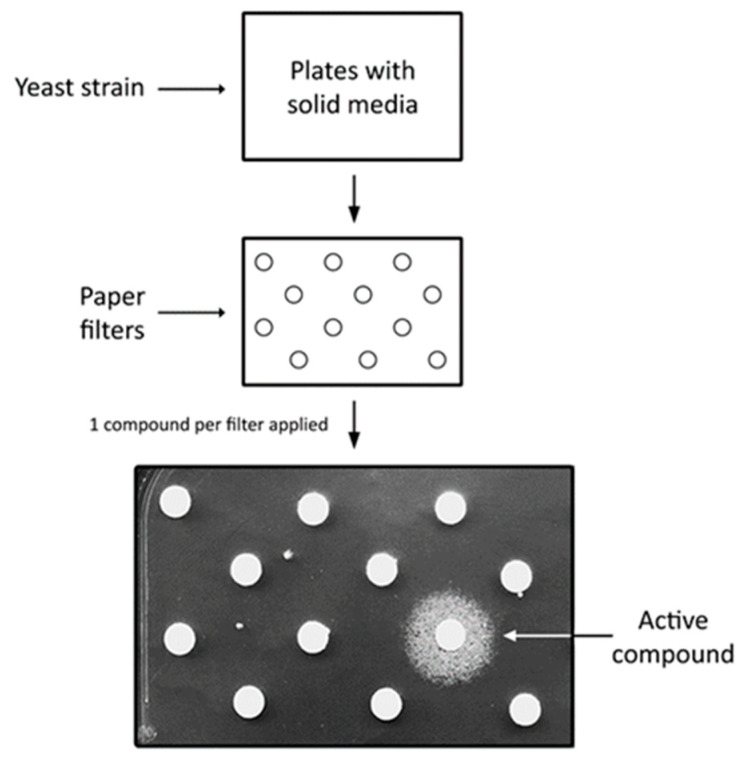
Scheme of a chemical suppressors screen using yeast. The presence of a growth zone around the paper filter indicates that an active compound has been spotted onto the filter. Figure adapted from Soczewka et al. (2020) [41].

Suppression could be achieved by treating a yeast model with biologically active chemical compounds. This kind of suppression is referred to here as chemical suppression, and it is analogous to drug intervention in humans. Drugs that suppress the effects of the modelled mutation could be identified by screening chemical libraries (Figure 1). However, a requirement of this approach is a convenient, reversible phenotype that enables easy identification of the active compounds among thousands of tested drugs. Yeast can also be used for studying a drug’s mechanism of action, similarly to genetic suppressors [15]. By searching for the yeast mutant in which an active drug becomes inactive, genes that are essential for the drug’s activity can be identified. Another strategy of searching for drug targets is to screen yeast knockout collection against the drug of interest. The idea behind this approach is that a yeast strain with deletion of a gene encoding a drug target is more sensitive to this drug in comparison to other yeast mutant strains [15].

Using yeast genetic and chemical screens has been fruitful in elucidating the pathogenesis of many diseases, and the findings in yeast models have been confirmed in higher eukaryotic disease models [33,35,36,37,38,39]. Most importantly, identified drugs were also active in human cell models [33,40].

## 3. *VPS13* Gene and Vps13 Protein in *Saccharomyces cerevisiae* Yeast

**Figure 2:**
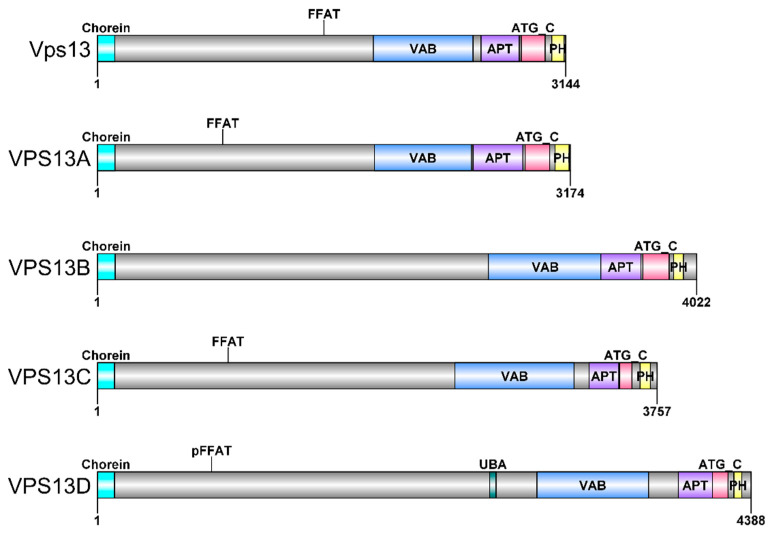
The schematic representation of the domain structure of VPS13 proteins. The domains of *S. cerevisiae* Vps13 and human VPS13A-D proteins; Chorein domain. VAB, Vps13 adaptor binding/WD40 domain; APT, APT1 domain; ATG_C, autophagy-related protein 2 C-terminal domain; PH, Pleckstrin homology-like domain; UBA, ubiquitin-associated domain; FFAT or pFFAT, two phenylalanine in acidic tract motif or phospho-FFAT motif. Presented with the use of DOG [43].

*VPS13* are evolutionarily conserved genes encoding VPS13 proteins; thus, it is possible to study them in various model organisms [13]. The majority of research was conducted using *S. cerevisiae* yeast, where one *VSP13* gene, encoding the Vps13 protein, was present. With a length of 3144 amino acid residues and a molecular mass of 358 kDa, Vps13 is one of the largest proteins found in yeast. It shows a complex domain structure, which is shared with human homologous proteins (Figure 2), and it contains several domains that are able to bind lipids and/or proteins. The domain structure and functions of VPS13 proteins were recently excellently reviewed [42].

Originally, *vps13*Δ was identified as one of the mutants in the screen for yeast strains defective in vacuolar protein sorting of the carboxypeptidase Y [44,45]. In further studies, more protein trafficking phenotypes have been reported for the *vps13*Δ mutant, including missorting of several Golgi apparatus proteases [46,47], vacuolar cargo receptor Vps10 [46], sorting adaptor Sna3 [48], vesicle membrane receptor Snc1 and flippase Neo1 [49]. Sorting defects in the *vps13*Δ mutant suggest the involvement of Vps13 in intracellular transport. Indeed, in vitro studies showed that Vps13, together in complex with calcium-binding centrin Cdc31, is required for Trans-Golgi Network (TGN) homotypic fusion and TGN to multivesicular body transport [50]. Next to the role in protein trafficking, Vps13 is involved in mitochondria functioning, since the *vps13*Δ mutant shows elevated transfer of mitochondrial DNA to the nucleus and enhanced mitophagy [51]. The *vps13*Δ mutant also exhibits defects in the actin cytoskeleton organisation and endocytosis [48], a process which highly depends on forces generated by the actin cytoskeleton [52]. Moreover, Vps13 interacts with actin [48] and is present in actin patches [53]. This suggests a role for Vps13 in actin cytoskeleton regulation. Another process in which Vps13 is involved is sporulation [46,54,55].

Recent studies strongly suggest that VPS13 proteins act as lipid transfer proteins at membrane contact sites (MCS)—zones of close proximity between the membrane of one organelle and the membrane of another organelle or the plasma membrane. MCS enable the direct exchange of small metabolites (lipids, ions and amino acids), signal transduction and enzymatic regulation in trans [56]. The N-terminal part of Vps13 from *S. cerevisiae* yeast was shown to bind phospholipids and transfer them between phospholipid bilayers in vitro [57]. Moreover, structural studies of the Vps13 N-terminal fragment obtained from *Chaetomium thermophilum* fungus revealed that it forms a long groove with hydrophobic and hydrophilic amino acid residues facing the interior and exterior sites, respectively. This groove architecture is suitable for solubilizing lipids, suggesting that Vps13 serves as a bridge between the membranes of different organelles and mediates bulk lipid flow [58,59]. It is unknown yet, how (and if) the specificity, rate and direction of the lipid transport are regulated in the bridge model [60]. The recent mechanistic model of Vps13 functioning was developed and reviewed by Leonzino et al. [61].

**Figure 3:**
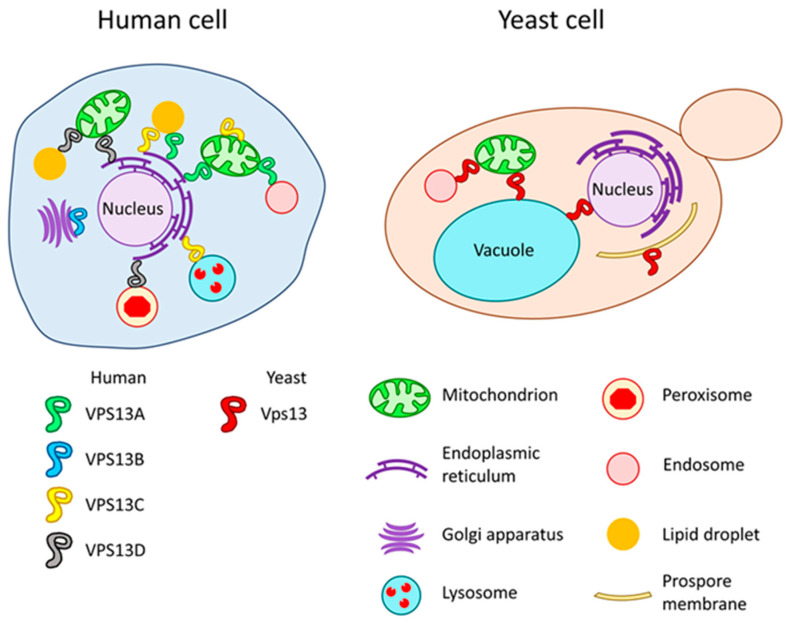
Localisations of VPS13 proteins in human and yeast cells. Shape of VPS13 proteins is based on electron microscopy pictures of yeast Vps13 protein [50].

Vps13 in yeast cells was found at the MCS between the vacuole and mitochondria; vacuole and nucleus; and endosome and mitochondria (Figure 3) [51,62,63]. As Vps13 is present in multiple sites, the regulation of Vps13 localisation is required. One way in which the localisation of Vps13 is achieved is the interaction of the VPS13 adaptor binding (VAB) domain with respective adaptors. The VAB domain contains a set of six repeated sequences which bind proteins with PxP motifs [64]. The PxP motif is present in Vps13-binding proteins including a sorting nexin Ypt35, mitochondrial outer membrane protein Mcp1 and meiosis specific adaptor Spo71. All of these proteins have been found to influence Vps13 localisation: Ypt35 targets Vps13 to endosomal and vacuolar membranes [64]; Mcp1 to mitochondria [65]; Spo71 to prospore membrane [66]. These adaptors compete to bind and recruit Vps13 to a desirable location, depending on the environmental conditions or state of the cell [64]. These aspects were comprehensively reviewed by Dziurdzik et al. (2021) [42]. Recently, C-terminal PH-like domain was shown to be a determinant of Vps13 localisation to TGN due to its ability to interact with Arf1 GTPase [67]. Vps13 localisation could also be influenced by lipid binding. Various domains of Vps13 were shown to bind to different signalling lipids, such as phosphorylated phosphatidylinositols and phosphatidic acid, and the specificity of binding differs for different Vps13 domains [48,50,67,68]. Interestingly APT1 domain binding to phosphatidylinositols was found to be regulated by calcium ions (Ca^2+^) [68]. The involvement of phosphatidylinositol-4-phosphate (PI4P) in regulation of Vps13 was also recently shown [55].

Research regarding Vps13 protein using the yeast model contributed to a better understanding of Vps13 functioning and gave background to recent groundbreaking studies regarding VPS13 proteins’ role in lipid transfer. It is one of the many examples of how yeast as a model of a eukaryotic cell improved the state of knowledge in the field of cell biology.

## 4. *VPS13* Genes and VPS13 Proteins in Human and Related Diseases

The human *VPS13* gene family consists of four members: *VPS13A*, *VPS13B*, *VPS13C* and *VPS13D* [69]. The length of the *VPS13* genes ranges from 208 to 864 kb, and exons number from 66 to 86 [69]. Expression is ubiquitous in various tissues, with some differences within splicing variants [69,70,71]. Mutations in the *VPS13* genes are associated with several rare neurological diseases. VPS13A-D proteins localize to various MCS, where it is possible that they all transfer lipids between membranes.

Mutations in *VPS13A* result in chorea-acanthocytosis (ChAc), an ultrarare neurodegenerative disease [70,71,72] affecting less than 1–5 individuals per 1 million [73]. In most cases, pathogenic mutations result in a premature stop codon, reading frame shift or disturbed splicing, causing the absence of protein; however, missense mutations have also been described [13,74,75,76,77]. ChAc is a progressive disease, and the first symptoms occur in early adulthood. Patients exhibit various dysfunctions of the nervous system such as movement disorders (i.e., chorea, dyskinesias and dystonia), epileptic seizures, peripheral neuropathy, dementia, psychosis and swallowing difficulties [73]. These symptoms are similar to those observed in HD or PD patients, and many patients could be misdiagnosed [78]. Recently, a case of a patient diagnosed with PD bearing the *VPS13A*-delin mutation was reported [79]. In addition to neurological signs, a characteristic feature of ChAc is the presence of acanthocytes in patients’ blood. Acanthocytes are erythrocytes with altered, spiked morphology, and may account for up to 50% of red blood cells in patients [73,80]. VPS13A protein localises to ER–mitochondria, ER–lipid droplets and endosome–mitochondria MCS (Figure 3), where in addition to a role in lipid transfer, it is involved in the maintenance of these MCS [57,81,82]. Disturbed formation of MCS could be the cause of some of the observed mitochondrial-related defects in *VPS13A* knockout cell lines, such as abnormal mitochondrial morphology, increased mitochondrial fission and reduced elimination of damaged mitochondria by mitophagy [81]. Other autophagy-related defects were observed in VPS13A-depleted cells, but they are rather more general, not specific to a particular type of autophagy [82,83]. This could be the result of decreased lysosomal degradation in the absence of *VPS13A*, probably caused by impaired processing of lysosomal hydrolases [82]. VPS13A is also involved in organisation of the actin cytoskeleton [84,85]. It interacts with β-actin and β-adducin proteins [86], which are part of the erythrocyte membrane cytoskeleton and have a role in synaptic functioning [87,88]. Moreover, depolymerisation of the actin cytoskeleton contributes to increased synaptic activity observed in neurons generated from induced pluripotent stem cells (iPSCs) derived from ChAc patients [89]. The altered anchoring of the membrane to the cytoskeleton could be related to higher activity of Lyn kinase observed in ChAc red blood cells and phosphorylation-induced perturbation of protein–protein interactions [90]. Significantly, enhanced Lyn kinase activity was also demonstrated in ChAc neurons [89]. Recently, the scramblase XK was identified as a binding partner of the VAB domain of the VPS13A protein [91]. Surprisingly, XK and VPS13A were found in a complex in membrane fractions [92,93], but XK recruited VPS13A from lipid droplets to subdomains of the ER, not to the plasma membrane [91]. This is an interesting finding, because both Vps13 and XK have been shown to be important for phosphatidylserine exposure at the outer leaflet of the plasma membrane after stimulation of cells with ATP [92]. Finding the interaction between VPS13A and XK is important because mutations in the *VPS13A* gene manifest clinically similarly to mutations in the *XK* gene causative of McLeod syndrome [76,94]. VPS13A is recruited to ER membranes by interaction of its FFAT motif with ER membrane proteins, VAPA and VAPB [57], and it has the potential to bind Golgi apparatus membranes as its C-terminal PH-like domain binds yeast Arf1 GTPase [67]. Moreover VPS13A can be recruited, by its APT1 domain, to endosomes in a Ca^2+^-dependent manner [68].

Mutations in *VPS13B* lead to Cohen syndrome—a disorder characterised by psychomotor retardation, intellectual disability, microcephaly and characteristic facial features [95,96]. Cohen syndrome is the only *VPS13*-related disease that is manifested already at birth. Similarly to ChAc, mutations in *VPS13B* predominantly result in the lack of functional protein [97]. VPS13B is a peripheral membrane protein of the Golgi apparatus (Figure 3) and is required for its integrity [98]. Localisation of VPS13B to Golgi is mediated by interaction with RAB6 [99], a GTPase regulating intra-Golgi transport and exocytosis [100,101,102]. VPS13B deficiency impairs protein glycosylation, a process occurring in Golgi apparatus [103]. This defect is thought to be a key factor contributing to disease pathogenesis [103]. Finally, VPS13B depletion, contrary to VPS13A, increases autophagic flux [104].

Mutations in *VPS13C* are associated with early-onset PD [105,106,107] and type 2 diabetes [108,109,110,111]. VPS13C protein is present at MCS. It was found in the ER–lysosome and ER–lipid droplets MCS (Figure 3), so the latter localisation is shared with VPS13A [57]. Another study showed that VPS13C localises at the outer mitochondrial membrane [112]. Loss of *VPS13C* function results in abnormal mitochondrial morphology, lower mitochondrial membrane potential and increased respiration rates [112]. Finally, VPS13C binds galectin-12, an adipocyte protein involved in adipocytic differentiation and lipolysis regulation [113,114,115]. Both proteins are upregulated during adipocyte differentiation and VPS13C is required for galectin-12 stability [113].

Mutations in *VPS13D* result in complex neurological disease, named *VPS13D* movement disorder. Patients exhibit movement defects (chorea, ataxia, dystonia), in some cases combined with intellectual disability [116,117,118]. Loss of *VPS13D* function causes reduced mitochondrial fission, resulting in defective mitophagy and impaired mitochondrial morphology [119]. Moreover, mitochondrial morphology, accompanied with lowered ATP production, was observed in patient-derived fibroblasts [117]. Recent studies showed that VPS13D localises to MCS. VPS13D, like VPS13A, was found at ER–mitochondria, but also at ER–peroxisomes MCS (Figure 3) [120]. The latter is most likely crucial for observed VPS13D function in peroxisomes biogenesis [121]. Another study revealed that VPS13D mediates contact sites between mitochondria and lipid droplets (Figure 3), and, together with the endosomal sorting complex required for transport (ESCRT), facilitates fatty acids transfer from lipid droplets to mitochondria in in vitro experiments [122].

The *VPS13A* gene, which is most similar to yeast *VPS13* [69], does not complement *vps13*Δ; therefore, the *vps13-I2749R* mutation mimicking *vps13A-I2771R* point mutation found in the ChAc patient was functionally analysed. This mutation causes amino acid residue substitution in the APT1 domain and abolishes Ca^2+^-dependent binding of this domain to selected phosphorylated phosphatidylinositols in vitro and changes Vps13 localisation in vivo [48]. Several other pathogenic *VPS13A-D* mutations were tested in the same way in yeast: *vps13A-L67P, vps13A-L1095P, vps13A-Y2721C; vps13B-N2993S, vps13C-W395C, vps13C-F444P, vps13C-L2789T, vps13D-L2900S, vps13D-N3521S, vps13D-D4107I, vps13D-A4210V* and *vps13D-R4228Q*, [10,11]. The VPS13 protein level and different phenotypes including CPY secretion and effect on sporulation were used as a measure of pathogenicity. Moreover, it was shown that some mutations causing substitutions in VAB domains result in disruption of adaptor binding in yeast Vps13, which gives insight to diseases pathogenesis [10,64].

## 5. Calcium Signalling as a Potential Target for Drug Intervention in *VPS13*-Dependent Neurodegenerative Diseases

The knowledge about VPS13 proteins is still not sufficient to develop a specific therapy for patients. To date, gene therapy is also not available. Only symptomatic treatment is used, and new methods to alleviate the symptoms are needed. The most efforts for treatment development are reported for ChAc. The deep brain stimulation is used to relieve movement problems [118], botulinum toxin to treat dystonia and selected drugs for seizure control [77]. Based on the finding that Lyn kinase is hyperactivated in red blood cells of ChAc patients and neurons [89,90], the inhibitors of this kinase were also tested. As Lyn kinase inhibition represents a potential treatment in ChAc to restore some neuronal function [89], the trial to experimentally treat 3 patients was conducted. Although a partial restoration of the actin cytoskeleton was observed in red blood cells, the lack of improvement of neurological symptoms was noted after six-month drug administration [123]. Further, studies on mice models revealed that the drug tested, dasatinib, is not able to cross the blood–brain barrier, but another potential kinase inhibitor, nilotinib, can do that and improve not only haematological but also neurological defects in mice [124,125]. Since the variety of potential treatments is very limited, there is a great need for new drug candidates which could be effective in ChAc patients.

To find therapeutic alternatives based on the understanding of cell biology, using model organisms is necessary. Several yeast models of *VPS13*-dependent diseases were constructed, which show various phenotypes [10,11,13,48,51] as described above. However, these phenotypes were not suitable for high-throughput screens. Finding novel growth phenotype of *vps13*, hypersensitivity to commonly used detergent sodium dodecyl sulfate (SDS), enable suppressor screens [126]. This gave better insight into the pathways which are disturbed in these cells which allowed for finding ways to overcome the observed defects.

Specifically, SDS hypersensitivity growth phenotype was used in a screen for multicopy suppressors of *vps13-I2749R* mutation, and several plasmids responsible for improved growth were isolated and analysed. One of them contained a fragment of the *MYO3* gene (*MYO3-N*) encoding N-terminal part (amino acid residues (aa) 1–775) of Myo3 protein (Myo3-N) [126]. Myo3 is a type I myosin protein involved in actin cytoskeleton organisation and endocytosis [127,128]. *MYO3-N* overexpression was also improving growth of the *vps13*Δ mutant in the presence of SDS. Importantly, the suppression by *MYO3-N* was not limited to SDS-hypersensitivity. *MYO3-N* corrected two other *vps13*Δ defects: depolarisation of the actin cytoskeleton and hypersensitivity to canavanine, the phenotypes implying defective endocytosis. Indeed, endocytic reporters (Las17, Myo3, Myo5 and Abp1 tagged with fluorescent proteins) that localise to sites of endocytosis, were shown to be present longer on the plasma membrane in the *vps13*Δ cells than in the wild-type, indicating delayed endocytosis. *MYO3-N* overexpression shortened patch lifetimes for Las17, Myo3 and Myo5. To conclude *MYO3-N* overexpression could partially improve endocytosis in *vps13*Δ alleviating canavanine hypersensitivity [126].

**Figure 4:**
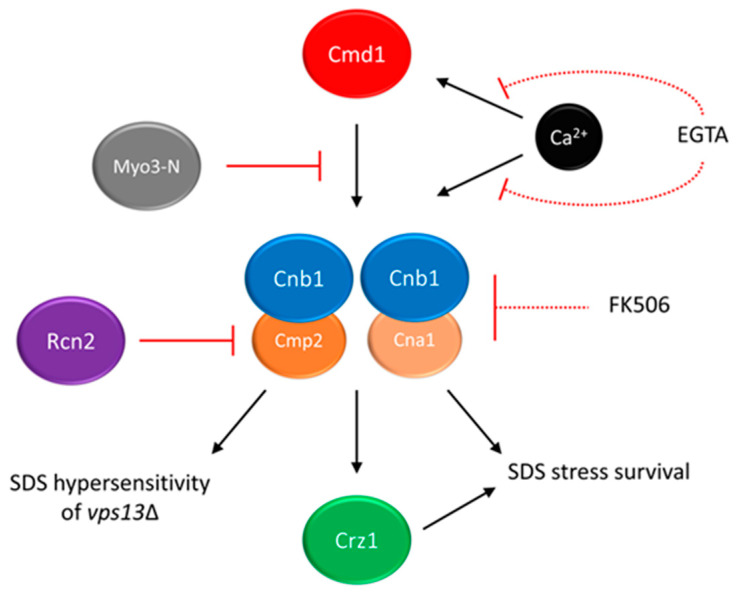
The calmodulin–calcineurin pathway and its impact on yeast growth in the presence of SDS stress. Partial inhibition of calcineurin (Cnb1 + Cna1/Cmp2) activity suppresses the SDS-hypersensitivity of *vps13*Δ cells. Multicopy suppressors *MYO3-N* and *RCN2* act by titrating calmodulin or blocking the activity of Cmp2 catalytic subunit, respectively. Chemical suppressors EGTA and FK-506 act by chelating the calcium ions required for calmodulin and calcineurin activities or inhibiting total calcineurin activity, respectively. As full inhibition of calcineurin activity is toxic in the presence of SDS stress, both chemical suppressors must be used in moderate concentration, indicated by dashed lines. Figure is based on results presented in Soczewka et al. 2019 and Wardaszka et al. 2021 [126,136].

In further analysis, the mechanism of *MYO3-N* action was elucidated. The suppressing *MYO3-N* fragment encodes myosin motor domain and a linker with two calmodulin binding motifs (IQ1 and IQ2). It is possible that suppression could be achieved by binding with calmodulin (Cmd1)—a conserved, calcium-binding protein mediating calcium signalling in cell [129]. To test this possibility, mutations disrupting both IQ motifs were introduced into *MYO3-N* and resulted *myo3-iq1/2* was not able to suppress *vps13*Δ and the interaction between Myo3-iq1/2 and Cmd1 was abolished. These results indicated that binding of calmodulin to Myo3-N is necessary for *MYO3-N*-based suppression of *vps13*Δ [126]. The hypothesis that titrating of calmodulin by Myo3-N may result in lowering the activity of one of its downstream targets, calcineurin, was formulated. In yeast, calcineurin consists of one regulatory (Cnb1) and one of the two catalytic (Cna1 and Cmp2) subunits [130,131,132] (see Figure 4). In response to changes in calcium concentration, calcineurin regulates gene expression via its target, the Crz1 transcription factor [133,134]. In fact calcineurin activity is higher in the *vps13*Δ cells than in the wild-type, and *MYO3-N* overexpression reduces it (Figure 4) [126]. Remarkably, the Cnb1 subunit, which is essential for calcineurin activity, is required for *MYO3-N*-based suppression of *vps13*Δ. This indicates that the suppression mechanism of *MYO3-N* relies on downregulating calcineurin activity. However, calcineurin must not be shut off completely (Figure 4). This result resembles a study which showed that in a yeast model of PD, moderate calcineurin inhibition reduces α-synuclein toxicity, and deletion or overexpression of genes encoding calcineurin subunits exacerbate it [135].

The identification of the *RCN2* gene as another suppressor of *vps13*Δ [136] is in line with the results described above. *RCN2* encodes Rcn2 protein, a negative calcineurin regulator [137,138]. Interestingly, Rcn2 binds preferentially to the Cmp2 calcineurin catalytic subunit in comparison to the Cna1 catalytic subunit. Analysis indicated that the N-terminal fragment of Rcn2 is necessary to reduce calcineurin activity, maintain the Rcn2 interaction with Cmp2 and to improve growth of *vps13*Δ (Figure 4). Moreover, the deletion of *CMP2* actually suppresses *vps13*Δ, contrary to *CNA1* and previously shown *CNB1* deletions, which negatively influence *vps13*Δ growth in the presence of SDS. The *CNA1* and *CNB1* are also required for *vps13*Δ suppression by *RCN2* [136] as for *MYO3-N*, indicating that *vps13*Δ suppression is achieved by reducing calcineurin activity related to Cmp2 catalytic subunit (Figure 4), while the activity mediated by Cna1 is crucial for SDS stress survival. Similarly, in mammals catalytic calcineurin subunits are differently expressed and regulated [139,140,141,142]. Knowledge about the specificity of various calcineurin forms and their role in disease molecular pathologies may contribute to development of novel and specific calcineurin inhibitors. Such specific peptide inhibitors of calcineurin would allow calcineurin activity involved in a disease to be downregulated without disturbing calcineurin-related processes required for healthy organism functioning. Various peptide inhibitors of human calcineurin were already studied [143,144]. This approach could, at least partially, eliminate side effects of general calcineurin inhibitors, such as FK-506 immunosuppressant and cyclosporin A.

As partial reduction of calcineurin activity was responsible for the suppression, it is possible that *vps13*Δ SDS-hypersensitivity could be mitigated by pharmacological calcineurin inhibition. Indeed ethylene glycol tetraacetic acid (EGTA), which limits the calcium availability required for calmodulin and calcineurin activity, and FK-506 (Figure 4) improved the *vps13*Δ growth, showing that it is possible to achieve *vps13*Δ suppression in a pharmacological manner [126]. Moreover, FK-506 was active only in very low concentration, and higher concentration caused toxicity. This is in line with genetic experiments and shows that basal calcineurin activity is required for SDS protection.

The proper functioning of calcium signalling is crucial for cells. Especially in neurons, the concentration and storage of Ca^2+^ ions in specific compartments are precisely controlled. Dysregulation of Ca^2+^ signalling is observed in ageing neurons and neurons affected by neurodegenerative diseases such as AD, PD and HD. Based on these findings, a hypothesis was formed that the dysregulation of Ca^2+^ signalling is the primary basis for the pathogenesis of neurodegenerative diseases. Indeed the changes in activity of calcineurin were observed in several neurological diseases [145,146], and its increased activity was reported in yeast models of PD [147]. Moreover, the store-operated calcium entry (SOCE), a mechanism of acquiring extracellular calcium triggered by Ca^2+^ depletion in the ER [148], was found defective in all major neurodegenerative diseases including AD, PD and HD [149]. The nature of impairment is characteristic for each of these diseases. While HD and PD are characterised by an excessive depletion of Ca^2+^ from ER stores in neurons, in AD neurons the ER is overloaded with Ca^2+^, as described in several recent reviews [149,150,151,152,153,154]. In addition, in ChAc patient-derived fibroblasts, levels of SOCE components, ORAI1 and STIM1 were reduced, and SOCE activity was downregulated [155]. Similar defects were observed in neurons generated from ChAc patient-derived iPSC, and these alteration in SOCE functioning, at least partially, contributed to neurodegeneration [156]. Despite enormous efforts, AD, PD and HD are still incurable, and only symptomatic relief drugs are available; research on effective treatment is still ongoing. One line of search for new therapeutic treatment is based on the Ca^2+^ signalling hypothesis of neurodegeneration. The effect of modulating the release of Ca^2+^ ions from ER storage sites and transport to cells was studied [157]. The relevance of Ca^2+^ signalling for neurodegenerative diseases further supports research focused on investigating it as a potential therapeutic target in ChAc patients.

## 6. Copper and Iron Homeostasis as a Potential Target for Treatment of *VPS13*-Dependent Neurodegenerative Diseases

Iron and copper are linked with neurodegenerative diseases with protein aggregation. AD, PD and HP are characterised by increased iron and/or copper levels in specific brain regions that are accompanied by cellular damage and oxidative stress [158,159,160,161]. Iron and copper interact with the amyloid precursor protein (APP) and its peptide derivative, amyloid beta (Aβ), both of which are involved in AD, and with α-synuclein which is involved in PD, while copper binds to huntingtin involved in HD [162,163]. It has been suggested that this interaction mediates protein aggregation and contributes to disease development [158]. Moreover, copper and iron stimulate the formation of advanced glycation end-products (AGEs), which are toxic and induce aggregation of proteins including those associated with the pathogenesis of AD [164]. However, a meta-analysis indicates a copper deficiency in the brain of AD cases [165], and most meta-analyses results suggest that overall and unbound copper are present in higher concentrations in serum samples of AD patients [166,167,168], suggesting copper dyshomeostasis. Based on these findings various metal chelators are under study for AD and PD in mouse models and in clinical trials [158,159,160].

There are not many reports on iron or copper dyshomeostasis in *VPS13*-dependent diseases or metal ion contribution in their pathogenesis so far [169]. Finding *FET4*, encoding iron transporter, and *CTR3*, encoding copper transporter, as suppressors of *vps13*Δ [41,170] provides a hint that metal disturbances could contribute to the pathogenesis of *VPS13*-dependent diseases and opens the possibility of discovering new drugs and drug targets aimed at the normalisation of these disturbances. For this purpose, a chemical suppressor screen (as depicted in Figure 3) of compounds from the Prestwick Chemical Library, a collection of 1280 drugs (most of which have been approved for use in humans), was performed and resulted in the identification of luteolin and tolcapone as *vps13*Δ chemical suppressors [41]. Luteolin is a natural compound belonging to the class of polyphenols called flavonoids which are plant secondary metabolites. They are associated with antioxidant, antiviral, antibacterial, anticancer and neuroprotective activities, and their therapeutic potential has been extensively studied [171,172,173,174]. The core of flavonoids is formed by two benzene rings connected with a heterocyclic pyranic ring. Their physico-chemical properties are determined by functional groups and their location in the flavonoid core [172,175]. Tolcapone, which is a drug used in the treatment of PD [176], has some structural similarities with luteolin, such as the location of benzene rings and hydroxyl groups on adjacent carbons. Moreover, both these drugs showed comparable activities when used in the same concentrations. These features indicate that luteolin and tolcapone could have the same mechanism of action. Tolcapone, however, exhibits serious adverse effects [177], while flavonoids are generally safe. The Prestwick Chemical Library contained one more flavonoid, ipriflavone, but it did not overcome the *vps13*Δ growth defect. To find out more about flavonoids as suppressors, a follow-up screen of the in-house library of approximately 50 natural compounds was performed [41]. An additional five drugs were identified as *vps13*Δ suppressors in this screen. Four of them—quercetin, pentaacetylquercetin, myricetin and fisetin—are flavonoids. The fifth compound, corilagin, belongs to tannins. In the collection of tested compounds, another flavonoid, kaempferol, was present but it did not improve *vps13*Δ growth. The only structural difference that distinguishes kaempferol from active flavonoids was that in the kaempferol structure, none of the hydroxyl groups are bound to adjacent carbon atoms. This criterion required for *vps13*Δ suppression by flavonoids was further confirmed in the structure–activity–relationship (SAR) analysis. The other structural criteria established during SAR analysis implicated that the heterocyclic pyranic ring must contain a double bond between C2 and C3 atoms and a carbonyl group, yet the ring itself does not necessarily have to be closed. A similar case is tolcapone, where the criteria regarding hydroxyl and carbonyl groups are met and benzyl rings are not connected by heterocyclic pyranic ring but by a carbonyl group.

**Figure 5:**
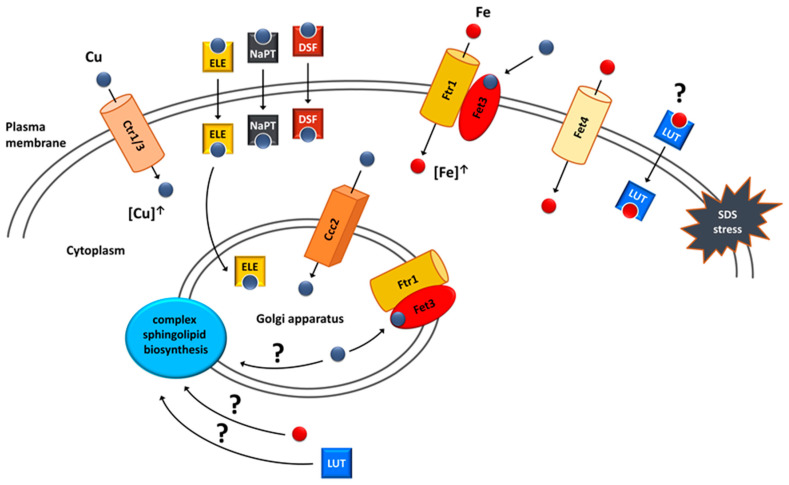
Suppression mechanisms of *vps13*Δ SDS hypersensitivity that involve copper or iron homeostasis. Suppression could be achieved by (I) overexpression of genes encoding copper transporters Ctr1, Ctr3 and Ccc2; (II) overexpression of the *FET4* gene encoding the Fet4 iron transporter; (III) treatment with copper ionophores (ELE—elesclomol; NaPT—sodium pyrithione; DSF—disulfiram); (IV) treatment with luteolin (LUT). Internalised copper is targeted to the Golgi apparatus where it is incorporated into the Fet3 oxidase, a part of the complex enabling high-affinity iron uptake. This complex is targeted to the plasma membrane via vesicular trafficking and increases the intracellular iron pool used for SDS protection. Extracellular copper can also be directly incorporated into Fet3 oxidase localised in the plasma membrane. It is possible that luteolin could act as iron ionophore. A potential target of copper, iron and luteolin action could be the biosynthesis of complex sphingolipids, which are important membrane components during SDS stress. Figure from Soczewka et al. (2021) [170] was supplemented and modified.

The structural criteria required for *vps13*Δ suppression overlaps with those previously described for flavonoids responsible for the antioxidant and metal chelation properties [178,179,180]. As *vps13*Δ was shown to be hypersensitive to cadmium [181,182], a heavy metal that causes oxidative stress, and all of the flavonoids that suppressed *vps13*Δ SDS hypersensitivity were also active when tested for *vps13*Δ cadmium hypersensitivity, a possible mechanism of their action could rely on their antioxidant properties. Contrary to this prediction, some of the tested flavonoids, such as kaempferol, did not improve *vps13*Δ growth, despite having higher antioxidant potential than the suppressing flavonoids [180]. This contradiction suggests that the alleviation of oxidative stress is a rather unlikely mechanism for flavonoid suppression of *vps13*Δ. Thus, it was hypothesised that metal chelation properties could possibly be important for *vps13*Δ suppression. This was supported by the fact, that one of the identified genes in the multicopy suppressor screen was *FET4* [41], which encodes a plasma membrane low-affinity iron transporter [183] (Figure 5). In addition, iron salts alone improved *vps13*Δ growth. These results highlight the importance of iron in the protection against SDS stress. The potential interaction between luteolin and *FET4* overexpression was tested and luteolin improved *vps13*Δ growth regardless of *FET4* overexpression, and no additive effects were observed [41]. This indicates that luteolin may involve iron in its mechanism and could act on the same process as *FET4* (Figure 5). The possibility was tested that *vps13*Δ could be defective in iron acquisition. However, measuring activities of pathways involved in response to low iron levels, as well as the growth test in the presence of ferrozine, an iron chelator limiting its bioavailability, indicate that iron acquisition in *vps13*Δ cells was not altered. Therefore, it remains to be tested directly whether luteolin acts by compensating iron deficiency in *vps13*Δ cells or by influencing other cellular process.

In line with these predictions, possible links between luteolin, iron and sphingolipids (Figure 5) were tested [41]. Sphingolipids are structural components of membranes with the highest enrichment in the plasma membrane, and they are involved in signalling and regulatory processes in cells [184,185]. They are especially abundant in cells of the nervous system, and alterations in their metabolism are implicated in neurodegenerative diseases such as multiple sclerosis and Sandhoff disease [186]. Previous studies showed that the *IPT1* gene, which encodes the inositol-phosphotransferase crucial for sphingolipid biosynthesis, is important for the SDS stress response in yeast [187]. Moreover, iron serves as a cofactor in enzymes of sphingolipid biosynthesis pathway [188], and luteolin was shown to increase ceramide level, one of the key sphingolipid, in cancer cell line [189]. This raised the hypothesis that both iron and luteolin could act on the sphingolipid biosynthesis pathway to suppress *vps13*Δ. In agreement with this, the *csg2*Δ strain, devoid of another enzyme from the sphingolipid biosynthesis pathway, was also hypersensitive to SDS. Moreover, deletions of both *ipt1*Δ and *csg2*Δ negatively interacted with *vps13*Δ, and luteolin and iron were able to suppress both of the double deletion strains [41]. The contribution of the sphingolipid biosynthesis pathway in *vps13*Δ phenotypes and the pathogenesis of *VPS13*-dependent diseases requires further study.

The primary finding that points to copper homeostasis as a potential suppression target was the identification of the *CTR3* gene in the screen for multicopy suppressors of *vps13-I2749R* and *vps13*Δ [170]. *CTR3* encodes the plasma membrane copper transporter [190]. Copper relevance for suppression was further confirmed by showing that overexpression of the *CTR1* gene, encoding the main plasma membrane copper transporter [191,192], as well as treatment with copper salts improved the *vps13*Δ growth (Figure 4). Moreover, one of the identified compounds as the chemical suppressor was disulfiram [170]—a copper ionophore used for alcoholism treatment, which is gaining interest as a potential anticancer drug [193]. Two additional copper ionophores tested also suppressed *vps13*Δ growth defect. One was elesclomol, a candidate anticancer drug [194,195], and the other was sodium pyrithione (Figure 5), which has an anion that is used as an antimicrobial agent [196]. The fact that identified multicopy and chemical suppressors are directly related to copper strongly suggests that increasing the cellular copper concentration could be one of the mechanisms of *vps13*Δ suppression. Copper-based suppression was not limited only to the SDS hypersensitivity phenotype. *CTR1* overexpression, as well as treatment with all three copper ionophores, mitigated *vps13*Δ growth defect in the presence of cadmium. When testing the suppressors for the canavanine hypersensitivity of *vps13*Δ, *CTR1* overexpression only slightly improved *vps13*Δ growth, whereas elesclomol was the only active drug. This finding was quite intriguing, because despite the fact that all of the suppressors were copper-related, their mechanisms of action in the cell could be different.

When elucidating potential mechanisms of copper action, the potential copper deficiency and its compensation by improved copper uptake was tested [170]. However, copper measurements and growth tests in the presence of copper chelator did not indicate any copper deficiency in *vps13*Δ. Moreover, in copper-abundant conditions, the copper level in *vps13*Δ cells was even slightly elevated compared to the wild type. Thus, SDS hypersensitivity of *vps13*Δ is not caused by copper deficiency, and suppression of this *vps13*Δ does not rely on compensating low copper level; thus, another mechanism of action must exist.

The mechanism standing behind copper-based suppression was related to iron uptake, and its importance for SDS protection was described above [41]. Copper is required for functioning of the high-affinity iron uptake system. Upon internalisation, copper is transported to the Golgi apparatus by Ccc2 ATPase [197,198], where it is incorporated into Fet3 oxidase, which, together with Ftr1 permease, forms a complex responsible for the high-affinity iron uptake [199,200]. After binding copper, this complex is transported to the plasma membrane, where it enables iron uptake. Increasing intracellular copper pool by *CTR1* overexpression would improve functioning of this system, resulting in increased iron content in the cell. Using a genetic approach, it was shown that this suppression mechanism is possible. *CTR1* did not suppress *vps13*Δ when *CCC2* or *FET3* were absent, which indicates that components important for the high-affinity iron uptake system are required for *CTR1*-based suppression [170]. To further highlight the relevance of the high-affinity iron uptake system, it was presented that *CCC2* acts as a *vps13*Δ multicopy suppressor; therefore, suppression could be achieved by increasing copper transport to the Golgi apparatus. Importantly, *CCC2* overexpression was not effective in the absence of *FET3*, which further shows that the high-affinity iron uptake system is essential for copper-based suppression.

The findings from genetic experiments pointing at iron uptake as targets of copper-related suppression were further supported by measurements of iron levels in yeast cells. The results show that the iron level in *vps13*Δ was lower than in the wild type, and it increased upon *CTR1* and, to some extent, *CCC2* overexpression [170]. The iron level in *vps13*Δ, however, was only moderately decreased in comparison to iron levels observed in the *fet3*Δ mutant. It was shown that the *fet3*Δ strain was less sensitive to SDS than *vps13*Δ [41]; therefore, lower iron levels could contribute to *vps13*Δ SDS hypersensitivity only to a low extent. Supplementing media with copper salts greatly increased intracellular iron levels in both wild-type and *vps13*Δ strains, which is another indication that increasing the intracellular copper pool increases iron uptake via the Fet3–Ftr1 complex [170]. Interestingly, upon copper supplementation, the difference in iron levels between the wild-type and *vps13*Δ strain was higher in comparison to the measurements for yeast cultivated without copper supplementation. This indicates that in the *vps13*Δ mutant, copper may not be utilised by the high-affinity iron uptake system as effectively as in the wild type.

It was also tested whether copper ionophores and copper, itself, act via the high-affinity iron uptake system. Neither the tested ionophore nor copper sulphate were active in *fet3*Δ *vps13*Δ, showing that their mechanism of action relies on iron uptake by the Fet3–Ftr1 complex. Interestingly, however, copper sulphate and elesclomol but neither disulfiram nor sodium pyrithione were able to improve the growth of the *ccc2*Δ *vps13*Δ double deletion mutant when higher concentrations were applied. Copper sulphate could rescue *ccc2*Δ *vps13*Δ by the direct incorporation of copper to Fet3 on the plasma membrane [170] (Figure 4). In this mechanism, there is no need for Ccc2, which delivers copper to the Golgi apparatus where copper binding to Fet3 occurs. In agreement with this, other works showed that the effects of *CCC2* deletion but not *FET3* deletion could be overcome by copper supplementation [197,198,199,201]. In the case of elesclomol, it was proposed that the lack of *CCC2* is overcome by its ability to effectively transport copper to the Golgi apparatus [170]. This is supported by previous studies in which elesclomol was able to correct defects observed in the yeast *ccc2*Δ mutant, and it was also effective in a Menkes disease model in which the *ATP7A* gene, a homologue of yeast *CCC2*, is mutated [202,203]. It is worth noting that elesclomol was the only ionophore that reduced *vps13*Δ hypersensitivity to canavanine, suggesting that its action is broader than the other tested copper ionophores.

The possibility that the mitochondrial electron transport chain (ETC) could be a target for copper-based suppression in *vps13*Δ was also tested, because all of the tested copper ionophores were linked with mitochondrial functioning, and their effectiveness in yeast models of mitochondrial diseases was proved [202,204,205,206]. However, the functional ETC was not necessary for elesclomol action in *vps13*Δ cells [170]. Next to the role in iron uptake, copper could potentially act by improving sphingolipid biosynthesis (Figure 4). Copper is involved in sphingolipid biosynthesis, because the yeast *ccc2*Δ mutant is defective in hydroxylation of complex sphingolipids [207]; however, the nature of this involvement is not known [208]. Therefore, this is another indication that the sphingolipid biosynthesis pathway is an interesting subject for investigation in higher eukaryotic cell models. Further studies will show whether copper homeostasis, copper-dependent iron uptake and sphingolipid biosynthesis are relevant for ChAc pathogenesis.

## 7. Conclusions and Future Perspectives

Several studies show that yeast could be successfully used in the modelling of human neurological diseases to better understand their pathology, find therapeutic targets and repurpose drugs. The SDS hypersensitivity phenotype of the yeast *vps13*Δ mutant was particularly useful for identifying several multicopy and chemical suppressors. Importantly, some of the suppressors, both multicopy and chemical, were found to act on the same pathways relevant for *vps13*Δ suppression. Analysis of these pathways revealed new defects present in Vps13-deficient cells, showing that they could be potential therapeutic targets.

The new findings show the importance of calcineurin for the functioning of *vps13*Δ cells. It was shown that the calcineurin activity increased in *vps13*Δ cells and the downregulating activity of Cmp2 catalytic subunit suppressed *vps13*Δ SDS hypersensitivity. Activity reduction could be achieved by overexpression of *MYO3-N* and *RCN2*, deletion of *CMP2*, or treatment with EGTA and FK-506. *MYO3-N* overexpression also corrected endocytosis and the defect in the actin cytoskeleton’s organisation in *vps13*Δ cells. These findings are especially interesting, because the actin cytoskeleton organisation is regulated by calcineurin [209]. Moreover, calcineurin inhibition ameliorated actin cytoskeleton depolymerisation induced by status epilepticus in mice [210]. Epileptic seizures are one of the symptoms of ChAc. In ChAc patient-derived cells, alterations in calcium homeostasis were described; however, their effect on calcineurin was not investigated. Analysing the calcineurin activity and its impact on the actin cytoskeleton in higher eukaryotic cell models may reveal if there are any calcineurin disturbances that could be related to ChAc pathogenesis, and if they are limited to specific calcineurin catalytic subunits. Perhaps, protein inhibitors of these specific catalytic subunits could be used for pharmacological intervention in the ChAc patients.

The other findings show the relevance of iron, copper homeostasis, copper ionophores and flavonoids for *vps13*Δ suppression. Overexpression of the *FET4* gene or treatment with iron salts improved *vps13*Δ growth. Increased iron import could also be achieved in a copper-dependent manner. Moreover, flavonoids may act using the same process as iron. However, the exact mechanism in which iron improves yeast growth in the presence of SDS is still not elucidated. One possibility is that both iron and flavonoids, and perhaps copper itself, improve sphingolipid biosynthesis in *vps13*Δ. Sphingolipids are important for SDS protection in yeast and for nervous system functioning in humans. Therefore, knowing that VPS13 proteins influence lipid transfer in cells, disturbed sphingolipid homeostasis may be an important factor in the pathogenesis of the *VPS13*-related diseases. Future studies are required to determine whether sphingolipid homeostasis is disturbed in human cells from patients and could be a target for therapeutic intervention.

Newly identified potential repurposable drugs which are effective in alleviating defects of yeast mutant cells, such as calcineurin inhibitors, flavonoids and copper ionophores, require intensive studies using available human cell and mouse models. HeLa *siVPS13A* [83], fibroblasts or red blood cells from patients and patient-derived neuronal cell models [211] together with a mouse model of ChAc [124] will help to answer the question of whether any of these drugs can be of use for intervention in ChAc and other *VPS13*-related diseases.

## Data Availability

Not applicable.
